# Deep Learning-Based Classification of Inflammatory Arthritis by Identification of Joint Shape Patterns—How Neural Networks Can Tell Us Where to “Deep Dive” Clinically

**DOI:** 10.3389/fmed.2022.850552

**Published:** 2022-03-10

**Authors:** Lukas Folle, David Simon, Koray Tascilar, Gerhard Krönke, Anna-Maria Liphardt, Andreas Maier, Georg Schett, Arnd Kleyer

**Affiliations:** ^1^Pattern Recognition Lab—Computer Science, Friedrich-Alexander-Universität Erlangen-Nürnberg, Erlangen, Germany; ^2^Department of Internal Medicine 3—Rheumatology and Immunology, FAU Erlangen-Nürnberg and Universitätsklinikum Erlangen, Erlangen, Germany; ^3^Deutsches Zentrum für Immuntherapie, FAU Erlangen-Nürnberg and Universitätsklinikum Erlangen, Erlangen, Germany

**Keywords:** artificial intelligence, arthritis, joint, bone, deep learning

## Abstract

**Objective:**

We investigated whether a neural network based on the shape of joints can differentiate between rheumatoid arthritis (RA), psoriatic arthritis (PsA), and healthy controls (HC), which class patients with undifferentiated arthritis (UA) are assigned to, and whether this neural network is able to identify disease-specific regions in joints.

**Methods:**

We trained a novel neural network on 3D articular bone shapes of hand joints of RA and PsA patients as well as HC. Bone shapes were created from high-resolution peripheral-computed-tomography (HR-pQCT) data of the second metacarpal bone head. Heat maps of critical spots were generated using GradCAM. After training, we fed shape patterns of UA into the neural network to classify them into RA, PsA, or HC.

**Results:**

Hand bone shapes from 932 HR-pQCT scans of 617 patients were available. The network could differentiate the classes with an area-under-receiver-operator-curve of 82% for HC, 75% for RA, and 68% for PsA. Heat maps identified anatomical regions such as bare area or ligament attachments prone to erosions and bony spurs. When feeding UA data into the neural network, 86% were classified as “RA,” 11% as “PsA,” and 3% as “HC” based on the joint shape.

**Conclusion:**

We investigated neural networks to differentiate the shape of joints of RA, PsA, and HC and extracted disease-specific characteristics as heat maps on 3D joint shapes that can be utilized in clinical routine examination using ultrasound. Finally, unspecific diseases such as UA could be grouped using the trained network based on joint shape.

## Introduction

Arthritis is defined as inflammation of articular structures. As such, it is heterogeneous condition comprising several different diseases like rheumatoid arthritis (RA) and psoriatic arthritis (PsA) (1, 2). Classification of arthritis remains highly challenging, as specific biomarkers are often lacking. Thus, while in some cases classification of arthritis is rather straight-forward, e.g., through the presence of anti-citrullinated protein antibodies (ACPA) (3) in RA or the presence of plaque psoriasis in PsA (4), many forms of arthritis remain undefined and are therefore termed as undifferentiated arthritis (UA) (5).

Arthritis usually imprints on the articular bone structure and leads to distinct change in the shape of the joint (6). This structural imprinting can be identified by conventional radiography searching for cortical breaks (erosions) or local excess of bone (spurs) on the periarticular cortical bone surface. However, such approach is notoriously challenging as it is based on the subjective interpretation of readers, positioning of the joint and the paucity of data taken up in two-dimensional radiographs. Hence, while architectural changes in the joints may allow distilling patterns that are associated with different forms of arthritis, the hardware and software instruments to detect such differences were not well developed.

Neural network gain increasing attention in various fields of medicine (7–9). Switching from classical machine learning methods to neural networks is motivated by the way, in which neural network operate (10). With appropriate preparation of the data and the choice of a suitable model they can identify structures in volumes that correlate with a certain defined patterns resembling a defined condition or disease. We have recently developed the instruments to apply neural networks to high-resolution peripheral quantitative computed tomography (HR-pQCT) scans on joints. HR-pQCT is currently the gold standard in visualizing bone *in vivo*, combining a three-dimensional approach with high spatial resolution. In arthritis patients, the technology is sensitive in detecting and quantifying surface changes that influence the shape of bone, such as erosions and bony spurs (6). Based on such HR-pQCT datasets from arthritis patients we have recently set up a convolutional supervised auto-encoder (CSAE) network that could reliably define the form of the articular bone without the need of human intervention (11).

## Methods

The conception of this study was to train and validate neural networks on the bone shape of metacarpophalangeal (MCP) joints from three well-defined conditions (HC, RA, and PsA) in a first step and then use these data to interpret the nature of undifferentiated arthritis in a second step.

### Patients and Controls

Seropositive RA, PsA, and UA patients as well as healthy controls were exported from a large HR-pQCT database at the Department of Internal Medicine 3 of the FAU (12, 13). Seropositive RA patients had to fulfill the ACR/EULAR 2010 criteria for RA (3) and had to be positive for anti-citrullinated protein antibodies (ACPA). PsA patients had to fulfill the CASPAR 2005 criteria for PsA (4). Healthy controls had to be free of any current or past signs of inflammatory arthritis, any severe and uncontrolled systemic disease, and had to have negative rheumatoid factor and negative CCP (12). Undifferentiated arthritis patients had to (i) have joint disease of more than 6 weeks at the time of HR-pQCT scan, (ii) not fulfill the criteria for classification of RA and PsA, and (iii) have no diagnosis of any other joint disease such as osteoarthritis, gout, or infectious arthritis. All patients and controls were evaluated by a rheumatologist (AK/GS/DS) and provided written informed consent. Approval to analyse the images was obtained from the Ethics Committee of the Friedrich-Alexander-Universität Erlangen-Nürnberg (approval number 324_16 B).

### Imaging

Scans were performed of the dominant hand MCP-2 head using an HR-pQCT scanner (Scanco Medical, Brütisellen, Switzerland). The acquisition of HR-pQCT scans closely followed a previously described procedure (14). Scanning was performed at an isotropic voxel size of 82 μm, an effective energy of 60 kVp, a tube current of 90 μA, and an integration time of 100 ms yielding a patient dose <8 μSv for 111 slices. Two hundred and three hundred slices were acquired depending on the patient's anatomy. Scans with motion grades higher than grade three were excluded from analysis (14).

### Classification Using Deep Learning

**Figure 1:**
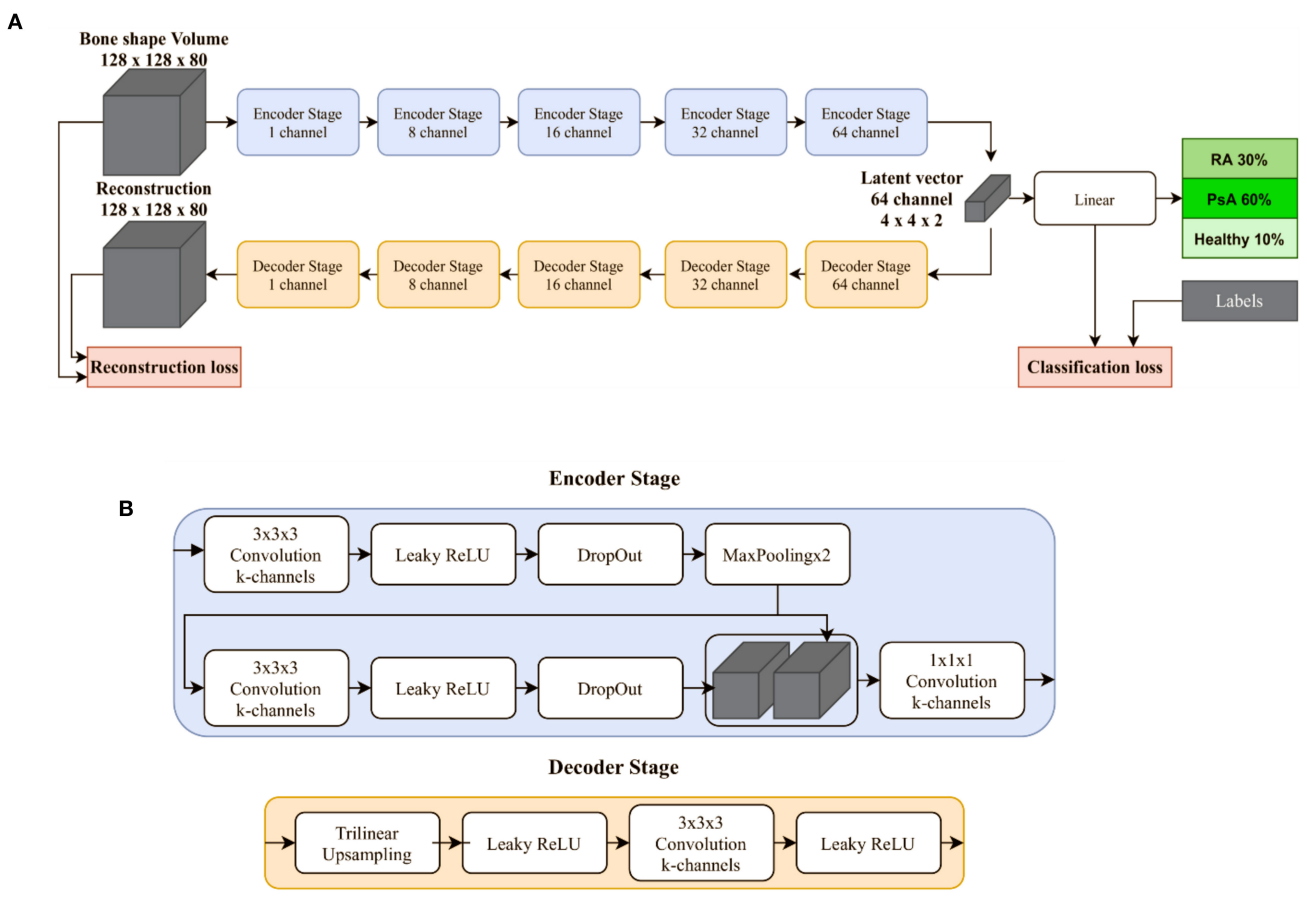
Deep learning model for the classification of joint shapes. **(A)** Proposed deep learning model termed Convolutional Supervised Auto-Encoder (CSAE) model consists of five stages each for the encoding and decoding branch. Stages closer to the linear classification layer have an increasing number of channels. **(B)** A single encoder consists of two 3 × 3 × 3 convolution followed by a Leaky ReLU activation function and three-dimensional dropout. Maximum pooling with a factor of two is used for down-sampling. The decoding branch is used to generate features in the bottleneck that are discriminative of the image.

Classification networks based on deep learning extract features present in images or volumes by applying mathematical operations that are referred to as *layers*. By the repeated extraction of features in a sequential manner and aggregation functions that reduce the dimensionality of the data, this results in a prediction for a given input volume. With the availability of large amounts of annotated volumes and suitable optimization techniques, neural networks can be trained to successfully classify volumes into predefined classes. Our proposed deep learning model (Figure 1) termed Convolutional Supervised Auto-Encoder (CSAE) model is based on previous supervised auto-encoder models by Le et al. (15). Details regarding the structure of the CSAE are provided on GitHub.

The loss function follows (15) and combines the reconstruction and the classification loss. However, instead of requiring the choice of two weights, we propose to just choose one weight: λ ∈ *R*. The input image $x_{i}\in R^{N}$ is compared with the reconstructed image ${\hat{x}}_{i}\in R^{N}$ using the mean squared error, while the predicted class ${ŷ}_{j}\in R^{M}$ is compared with the annotation $y_{j}\in R^{M}$ using cross entropy:


$$\begin{array}{l} L_{total}=\left(1-\lambda \right)\frac{1}{N}\overset{N}{\underset{i=1}{\sum }}{\left(x_{i}-\hat{x_{i}}\right)}^{2}+\lambda \frac{1}{M}\overset{M}{\underset{j=1}{\sum }}\hat{y_{j}}\log\left(\hat{y_{j}}\right)\\ \;+\left(1-y_{j}\right)\log\left(1-\hat{y_{j}}\right) \end{array}$$

### Experimental Setup

In our previous work, the second metacarpal bone head was segmented yielding a binary mask indicating the location of this region (11). This mask is a dense prediction of the bone region and does not preserve the internal microstructures of the bone. Based on this bone mask, a sub-region of the scan is extracted with the second metacarpal bone head in the center of the region. Subsequently, the cropped scans are resized to a uniform extent of 128 × 128 × 80 voxels. Finally, for each scan, the intensities are normalized by the subtraction of the mean value of the training set divided by the standard deviation of the training set defined during the five-fold cross-validation. These pre-processing steps yield two volumes of equal extent: The sub-region of the HR-pQCT scan, precisely, the second metacarpal bone head, and the corresponding bone mask indicating the region of the bone voxel-wise. For the classification task, in addition to the HR-pQCT sub-region inputs, we also investigated the performance of the model using only the bone mask extracted by the segmentation model described in reference (11). This way the model prediction is entirely based on the volumetric shape of the bone. Additionally, as a third input representation, bone mask and sub-region were combined using voxel-wise multiplication (Supplementary Figure 1). Five-fold cross-validation was used throughout all our experiments and the data split was performed on a patient level to avoid the presence of patients in the training and test dataset. To avoid a bias of the networks toward a more frequent class in the dataset, the loss function was weighted during training inversely proportional to number of classes of the specific case. Interpretability remains a valid concern when moving toward application of deep learning-based approaches. To increase the interpretability of our method we make use of the guided back propagation method (16). Using the classification label for a specific scan, this method allows the visualization of the voxels in the scan that contributed most to the decision of the model. Throughout the work we refer to those visualizations as topographical heat maps. In the second step of our approach, we applied the trained neural network to undifferentiated arthritis. The interpretation of those cases is based on a threshold of the network's certainty for each prediction. To remove uncertain predictions, probabilities smaller than 75% are disregarded thereafter similarly to Bressem et al. (17).

### Statistical Analysis

The measure for performance of the classification network was the area under the receiver operator curve (AUROC) ranging from zero to one, where one would be a perfect classifier. As our experiments contained three classes, the AUROC was calculated in an all-versus-rest fashion for each class. The F1 score represents the balanced mean of precision and recall (i.e., sensitivity and specificity) and ranges from 0 to 1, where one is the optimal value. A threshold of 50% was used for the sensitivity and specificity analysis. Additionally, we report the positive likelihood ratio for RA and PsA and interpret them as described by McGee (18).

## Results

### Patient Cohort

**Figure 2:**
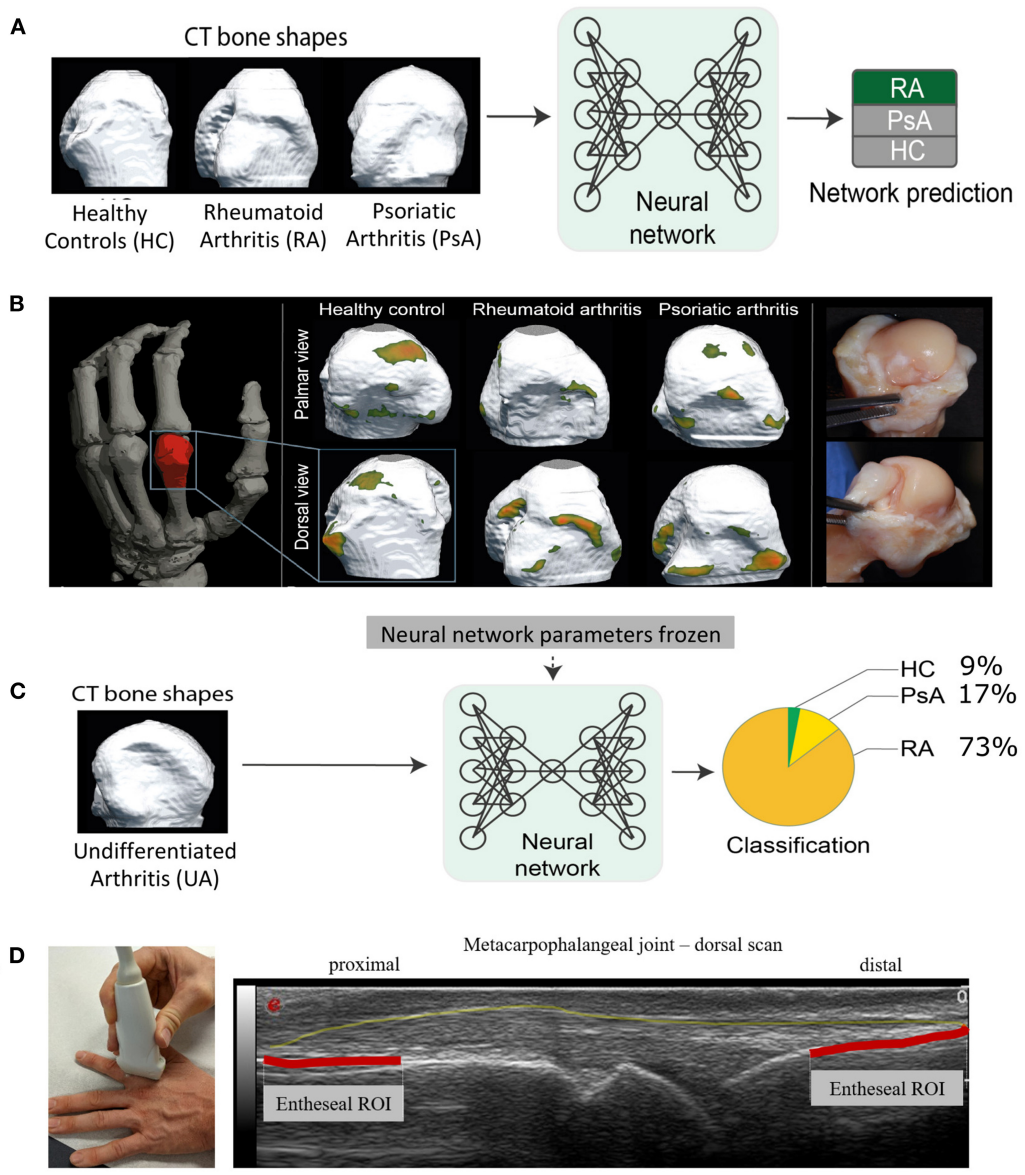
Training and validation of the neural network, visualization of the regions influencing the networks decisions and application of network to undetermined arthritis cases. **(A)** Training and validation of the neural network using the three-dimesional articular bone shape (assessed by high-resolution peripheral computed tomography) of defined conditions such as rheumatoid arthritis (RA), psoriatic arthritis (PsA), and healthy controls (HC). **(B)** Left: Location of the measurement region (red) of high-resolution peripheral computed tomography (CT) scans as data source; center: Three different segmentation bone masks with the respective heat maps from healthy controls as well as RA patients and PsA patients; each patient segmentation mask is shown in the palmar view (top row) and in the dorsal view (bottom row); right: Preparation of anatomical specimen to correlate to heat maps detected by the neural network with anatomical regions. **(C)** Application of the neural network using undifferentiated arthritis patients to classify them into either RA, PsA, and HC according to the neural network defined in **(A)**. **(D)** Ultrasound image, dorsal scan of a healthy metacarpophalangeal joint. Here, we illustrate the transfer of our findings to arthrosonography. The outline of the capsule is marked yellow. The articular entheseal regions are marked in red. Based on the findings of the neural network, alterations of these articular entheseal regions (red) are specific for PsA and should be paid attention in clinical routine, especially in patients who are suspected for PsA. Patients provided written consent to the depiction of their images.

In total, 932 volumetric HR-pQCT scans from 617 patients and healthy control were available after applying the exclusion criteria (192 scans removed due to high motion artifacts). An overview of the characteristics of the patients and the controls is shown in Table 1. These scans were used to train and validate the neural networks based on the segmentation bone masks representing the cortical bone surface of the articular bone (Figure 2A).

**Table 1:** Patients and controls.

		**Healthy controls**	**Rheumatoid arthritis**	**Psoriatic arthritis**	**Undifferentiated arthritis**
Number of scans		173	434	261	64
Number of patients		158	225	164	64
Age (ys; mean ± SD)		45 (±15)	55 (±11)	52 (±11)	59 (±12)
Sex (N; f/m)		88/70	155/70	86/78	31/16
Disease duration (ys; mean ± SD)		0	10 (±9)	6 (±5)	4 (±5)
ACPA positivity		0	100.00%	2.75%	0.00%
RF positivity		0	72.33%	6.66%	16.56%
Treatments (Numbers, ever)	csDMARDs	0	223	160	64
	bDMARDs	0	141	128	48
	tsDMARDs	0	39	12	8
Treatment duration (ys; mean ± SD)	csDMARDs	0	3.22 (±3.00)	2.36 (±2.22)	2.57 (±2.66)
	bDMARDs	0	3.79 (±3.21)	3.11 (±2.36)	3.11 (±3.04)
	tsDMARDs	0	1.10 (±0.82)	0.54 (±0.39)	1.14 (±0.79)

*Ys, years; csDMARDs, conventional synthetic disease modifying anti-rheumatic drugs; bDMARDs, biologic disease modifying anti-rheumatic drugs; tsDMARDs, targeted synthetic disease modifying anti-rheumatic drugs; ACPA, Anti–citrullinated protein antibody; RF, Rheumatoid factor; n/a, no data available*.

### Neural Network-Based Disease Characteristic Heat Maps on 3D Joint Shapes

Hotspots, regions that typically yielded in the greatest attention of the neural network (Figure 1), were those with high curvatures in the bone mask independent of the associated diagnosis (Figure 2B). Heat maps of regions that were critical for classifying diseases were those related to erosions in the bare area and osteo-proliferative changes in the ligament/capsule insertion sites (Figure 1B).

### Neural Networks Differentiating the Structure of RA, PsA, and Healthy Controls

We first applied the CSAE model based on the segmentation bone mask to differentiate HC, RA, and PsA (Table 2). Area under the receiver operator curve (AUROC) were 82% for HC, 75% for RA and 68% for PsA for discriminating between HC, RA, and PsA. A precision of 59% and recall of 58% were achieved. When using the HR-pQCT sub-region as an input to differentiate HC, RA, and PsA we received AUROC of 76% for HC, 75% for RA and 71% for PsA. This corresponds to a precision of 56% and a recall of 56%. Combined input of bone mask and HR-pQCT sub-region reached an AUROC of 78% for HC, 74% for RA, and 67% for PsA with a recall of 53% and a precision of 55%. The highest F1 score (19), resembling the highest balanced mean of precision and recall, was achieved by the HR-pQCT sub-region input with 58% followed by the segmentation bone mask with 57%, and the combined representation with 55%. The corresponding confusion matrices are shown in Supplementary Figure 2 for all different inputs.

**Table 2:** Classification results of the CSAE neural network for different input representations visualized in Supplementary Figure 1.

**Input representation**	**AUROC**			**F1 Score**	**Precision**	**Recall**
	**HC**	**RA**	**PsA**			
HR-pQCT Sub-region (#1)	76.24% (±3.44)	75.18% (±4.97)	71.34% (±4.31)	58.86% (±2.21)	56.98% (±0.21%)	56.76% (±1.92)
Segmentation Bone mask (#2)	82.38% (±4.44)	75.39% (±3.41)	68.29% (±5.05)	57.86% (±4.02)	59.20% (±1.40)	58.60% (±2.20)
HR-pQCT Sub-region and Bone mask (#3)	78.69% (±6.28)	74.89% (±2.55)	67.76% (±3.76)	55.53% (±4.40)	55.80% (±2.10)	53.21% (±0.81)

*CSAE, convolutional supervised auto-encoder; HR-pQCT, high-resolution peripheral quantitative computed tomography; AUROC, area under the receiver operator curve; F1 score represents the balanced mean of precision and recall (i.e., sensitivity and specificity); RA, Rheumatoid arthritis; PsA, Psoriatic arthritis; HC, Healthy control*.

Positive likelihood ratios for the detection of RA were 2.5 (±0.25) and 1.6 (±0.26) for PsA. Thus, a positive test result would lead to an increase of the probability of RA of about 17.4%, and about 8.9% for PsA.

### Classification of Undifferentiated Arthritis

We then applied the CSAE network to HR-pQCT data from patients with undifferentiated arthritis to classify them as RA or PsA (Figure 2C). The vast majority of the 64 undifferentiated arthritis patients (73%) were classified as “RA” (*N* = 47). The remaining patients were classified as “healthy” (9%, *N* = 6), while 17% (*N* = 11) were classified as “PsA” by the CSAE network. All patients classified as “PsA” by the neural network were receiving treatment with NSAIDs and most of them (86%) were under treatment with TNF inhibitors.

## Discussion

In this work, we developed a new model for classifying arthritis based on the shape of articular bone. We were able to train and validate the CSAE neural network to identify structural patterns in the hand joints in defined conditions such as RA, PsA, and HC. This was based on the detection of disease specific features visualized as heat maps by the NN. Followingly, the network revealed promising likelihood ratios to differentiate the shape patterns of bone between RA patients, PsA patients, and HC. Interestingly, the dense bone mask input for the neural network was superior to the subregion HR-pQCT scan input for the classification task, suggesting that the outer contour of the bone was sufficient for the network. Furthermore, we were able to apply this network to UA allowing classifying this heterogeneous group of patients. While most patients with UA clustered into seropositive RA, a smaller fraction was classified as either PsA or HC.

Neural network-based approaches in arthritis are in its infancy. Some recent efforts used electronic health records to train neural network in predicting clinical disease activity or differentiating RA from non-RA (20, 21). With respect to imaging, machine learning was applied to MRI scans of the hips of patients with and without osteoarthritis. The authors could show a dependency between the radiographic score of osteoarthritis and the shape of the femoral bone (22). Neural networks have also been used for the detection of radiographic sacroiliitis achieving high agreement with reference judgement (17). More traditional machine learning approaches have also been applied to hand radiographs to differentiate between RA and other conditions as well as to correlate ultrasound images with RA disease activity (23–25).

In our work, we analyzed a large set of well-annotated data, which allowed us to validate the model thoroughly. Furthermore, we refined neural networks as compared to the earlier works using multiple convolutional layers (15). In addition, we were able to project and thereby confirm the relevant regions, which were visualized by the heat maps. Hence, the inherent black box characteristic of neural networks could be minimized by the visualization of regions influencing the networks prediction that were on par with previous studies. The approach to train networks with precise datasets from well-defined diseases, such as RA and PsA, as well as the integration of HC in such networks provides the opportunity to test less understood and potentially heterogeneous conditions, such as in our case UA. Hence, such an approach may facilitate the classification of arthritis in the future. Besides classification of arthritides, neural networks automatically analyzing the shape of the bone, could also be used for predicting the course of the disease either at time of diagnosis or at start of treatment. In addition, neural networks could be applied to analyse data from patients at very early phases of disease, such as people at-risk for developing RA or those with psoriasis at risk to develop PsA to define patterns that also found in clinical disease and thus render patients at higher risk to progress to clinical disease (26, 27). In a follow-up study, this hypothesis could be analyzed with a dedicated cohort of patients in the early phase of the disease.

One limitation of this work is the fact that the network is so far only trained on RA, PsA, and healthy controls. Therefore, the network currently only allows clustering into these three categories, meaning that the network is trained for finding the best match of an unknown condition to either one of these three conditions. Other disease categories may not share these patterns and may require additional training of the network. The focus on hand joints may be considered as another limitation, however, RA, PsA and also undifferentiated arthritis most often affect the hand and therefore this approach is pragmatic, though not inclusive. Oligo- and polyarthritis information and LEI would be helpful to further characterize the patient cohort but was unfortunately not available.

On clinical relevance: Naturally, HR-pQCT is not widely available in clinical routine, which marks a major limitation of our technique. However, we have discovered disease-specific characteristics via visualization of the attention of our neural network as heat maps. For example, in PsA, the corresponding hotspots are located in the area of the articular entheses which have been described as articular-entheseal organs previously (28, 29). Thus, we emphasize to pay attention to these apparently very specific bone alterations especially in this region using other imaging modalities, which are broadly available such as ultrasound. Interestingly, these articular entheseal regions have not yet been in the focus especially in the workup of PsA. Therefore, where psoriatic arthritis should be ruled out, we recommend examining this region by ultrasound. Since sonography has a very high resolution, especially at the bone surface, we assume that the changes are also comparably visible as shown in our concept in Figure 2D. We seek to validate our finding in a follow-up ultrasound study.

Briefly, the combination of HR-pQCT and neural networks allowed us to derive important disease-specific characteristics that can be examined using sonography in clinical practice.

In summary, these data indicate that neural networks trained on the shape of joints can discriminate between the two main forms of inflammatory arthritis, RA and PsA, as well as healthy controls. Furthermore, if such networks are fed with the data from less well-defined conditions, such as UA, they allow assigning and clustering such conditions, which in the future and with ongoing refinement of networks could improve disease classification, i.e., in the absence of classical biomarkers.

The new findings discovered here should be specifically investigated using established and widely available imaging methods such as ultrasound.

## Data Availability Statement

The original contributions presented in the study are included in the article/Supplementary Material, further inquiries can be directed to the corresponding author.

## Ethics Statement

The studies involving human participants were reviewed and approved by Ethikkommission der Friedrich-Alexander-Universität Erlangen-Nürnberg. The patients/participants provided their written informed consent to participate in this study.

## Author Contributions

LF designed and performed the experiments. DS assessed patient diagnosis and performed CT scanning. KT validated the statistical methods. GS assessed patient diagnosis. AK assessed patient diagnosis, performed CT scanning, and designed the experiments. All authors reviewed the manuscript.

## Funding

This work has been supported by the German Research Council (CRC1181, CRC1483, FOR2438, and PANDORA FOR2886), the German Ministry of Science and Education (Project Mascara; grant 01EC1903A), the European Union (project ERC-Syn 4DNanoscope), the Innovative Medicine Initiative (RT-Cure and Hippocrates), and the emerging field initiative (project 4 Med 05 MIRACLE) of the University Erlangen-Nürnberg.

## Conflict of Interest

The authors declare that the research was conducted in the absence of any commercial or financial relationships that could be construed as a potential conflict of interest.

## Publisher's Note

All claims expressed in this article are solely those of the authors and do not necessarily represent those of their affiliated organizations, or those of the publisher, the editors and the reviewers. Any product that may be evaluated in this article, or claim that may be made by its manufacturer, is not guaranteed or endorsed by the publisher.
