# Supplementary material for: Deep Learning-Based Classification of Inflammatory Arthritis by Identification of Joint Shape Patterns—How Neural Networks Can Tell Us Where to “Deep Dive” Clinically
Source: Front Med (Lausanne). 2022 Mar 10;9:850552. doi: 10.3389/fmed.2022.850552 (PMC8960274; doi:10.3389/fmed.2022.850552)

**Supp. Figure 1. Preprocessing steps for the neural network.**

The three different inputs to the classification network are generated using the original HR-pQCT scan (only one slice visualized). The first representation is generated by cropping the MCP2 from the whole scan. The second representation is the result of the segmentation network applied to the sub-region. Finally, the third representation is generated by multiplying the first two inputs voxel-wise.


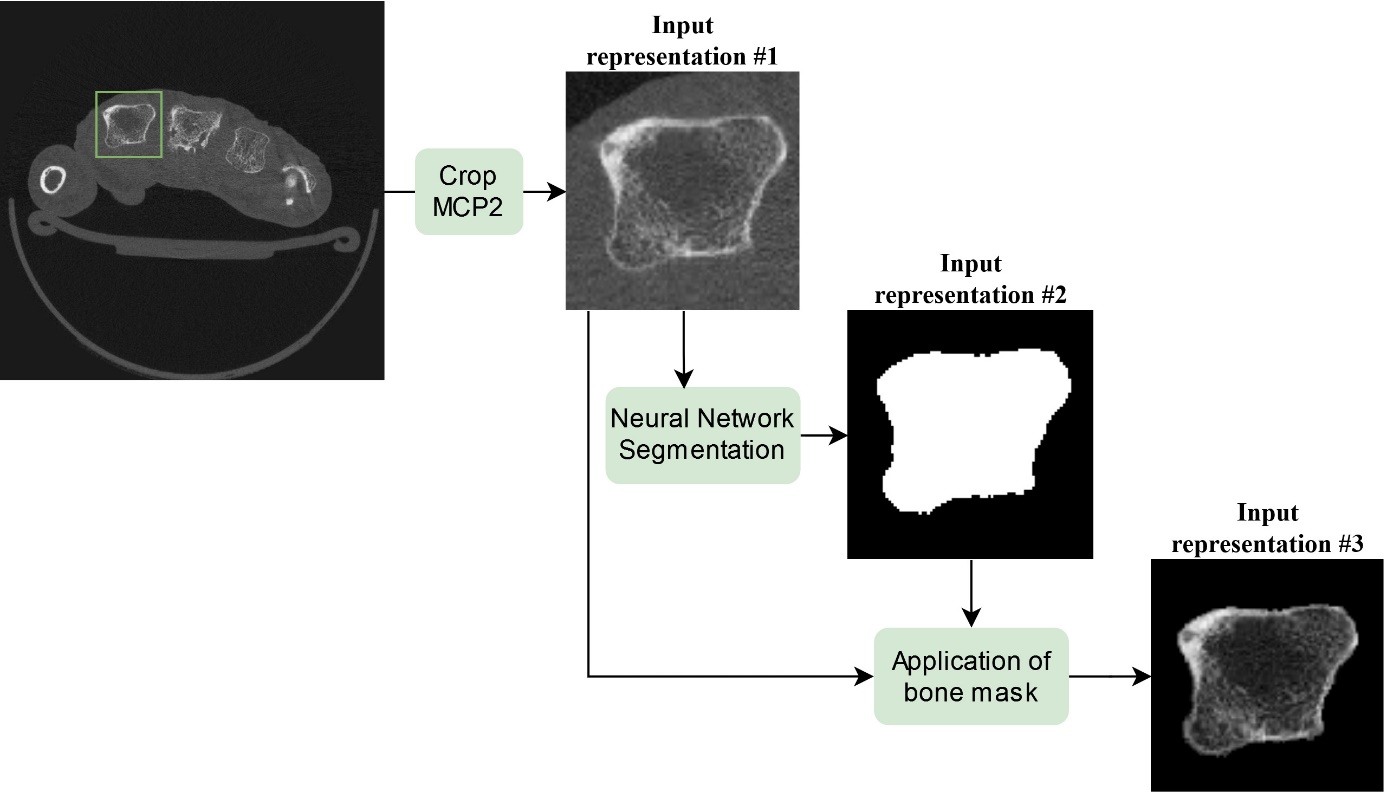


**Supp. Figure 2. Confusion matrices for the different neural networks**

For each input representation, the confusion matrix resulting from the five-fold cross-validation is represented. 0: Healthy control, 1: Rheumatoid arthritis, 2: Psoriatic arthritis. (A) HR-pQCT subregion input, (B) Segmentation bone-mask, and (C) Combined representation.


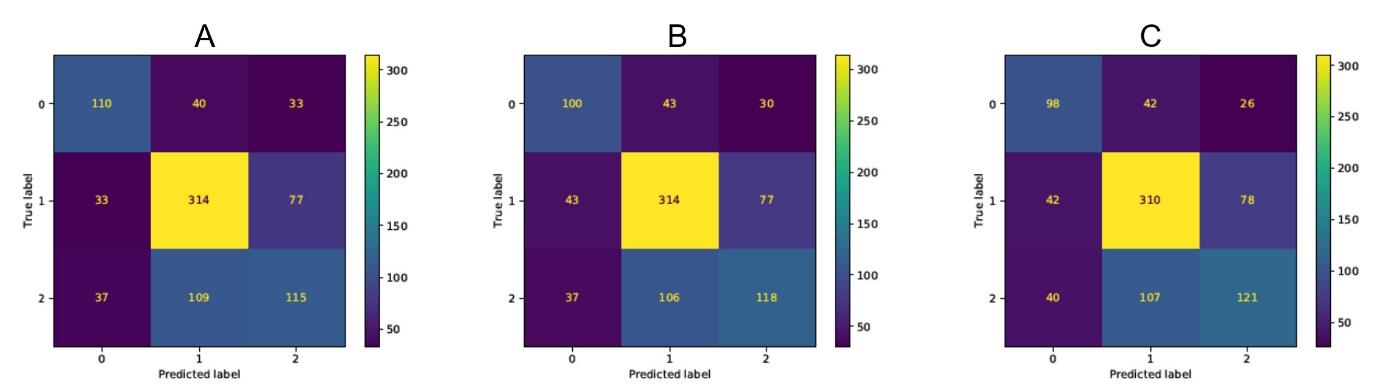

Supplement: Supplementary file 1 [file Data_Sheet_1.docx]
